# Warm-up cognitive activity enhances inhibitory function

**DOI:** 10.1371/journal.pone.0206605

**Published:** 2018-10-29

**Authors:** Kyoko Hine, Yuji Itoh

**Affiliations:** 1 Department of Information Environment, Tokyo Denki University, Chiba, Japan; 2 Department of Psychology, Keio University, Tokyo, Japan; Bournemouth University, UNITED KINGDOM

## Abstract

Society is aging worldwide. To promote the health and quality of life in elderly people, we must find a way to enhance and improve cognitive function, especially inhibitory function that suppresses inappropriate behaviors. Previous studies have indicated that warm-up cognitive activities enhanced the activation of appropriate behavior. As with the activation of appropriate behavior, inhibitory function is expected to be enhanced by a warm-up activity, although this theory has not yet been directly tested. Here, we investigated whether inhibitory function in a task was enhanced by a warm-up activity. We used a Navon task as a warm-up activity, in which reading small letters (local Navon task) required inhibitory function more than reading a large letter (global Navon task). The Stroop task was used as the subsequent task. Our results showed that the accuracy of the Stroop task after the local Navon task was higher than after the global Navon task. This outcome suggests that inhibitory function in the Stroop task was enhanced by the local Navon task, which was an inhibitory warm-up cognitive activity. Moreover, this study contributes to the development of new techniques of cognitive training to prevent the decline in inhibitory function during aging or other clinical scenarios, such as attention-deficit/hyperactivity disorder (ADHD).

## Introduction

Worldwide, society is aging rapidly, especially in developed countries. One serious problem in an aging society is the increase in the number of older people who have trouble carrying out their daily activities because of declining cognitive function. For example, the number of senior drivers who cause traffic accidents has been dramatically increasing in recent years in Japan because of the decline in cognitive function [1]. To enhance the quality of life of the elderly, we have to preserve and enhance their cognitive function.

Some previous studies have reported that inhibitory function is one of the cognitive functions that declines with aging [2]. Inhibitory function is a cognitive process that suppresses inappropriate thoughts or behaviors [3]. A decline in inhibitory function has been suggested to interfere with appropriate behaviors and facilitate inappropriate behaviors [4]. For instance, a driver mistakenly pushing down on the gas or brake pedals occurs due to a failure of the inhibition of inappropriate behaviors [5]. Therefore, to prevent such accidents, preserving and enhancing inhibitory function is crucial, although there are few reports about how to preserve or enhance inhibitory function [6]. Do we have any methods to strengthen inhibitory function?

**Fig 1:**
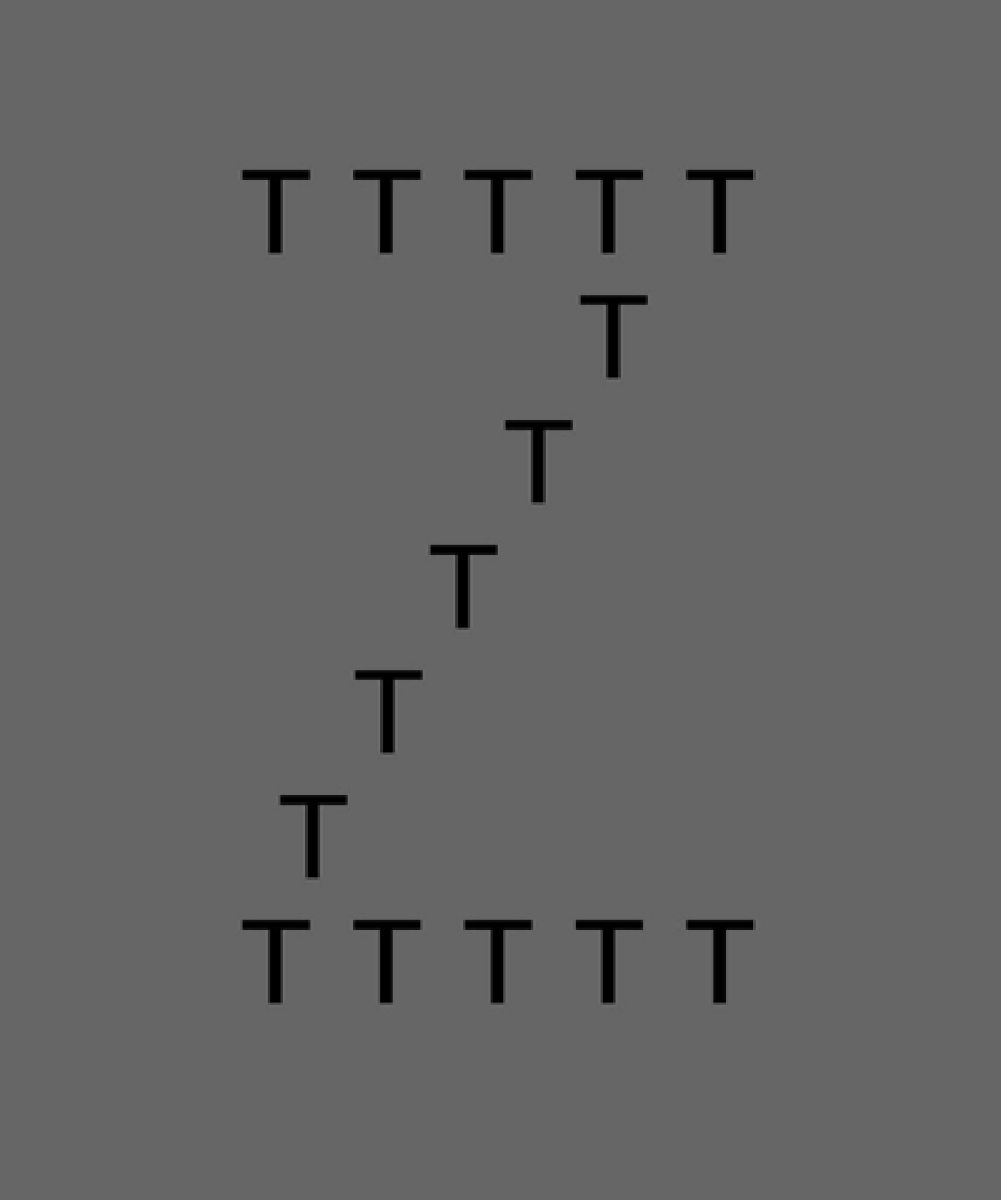
A sample of a Navon figure.

Previous studies have indicated that warm-up cognitive activities enhance the activation of appropriate behaviors or thoughts [7–12]. Hine and Itoh examined whether a warm-up activity enhanced performance in a subsequent facial memory task [7]. The appropriate behavior in the facial memory task is seeing a whole face [12–16]. In their study, the performance of the facial memory task was improved after a warm-up activity in which participants were required to read a large letter that was composed of small letters (e.g., the correct answer is “Z” in Fig 1). Therefore, these results suggested that the appropriate behavior in the facial memory task was enhanced by the warm-up activity. The effect of the warm-up activity was found in several experimental contexts of appropriate behaviors or thoughts, such as memory retrieval [7, 8], creativity [17], and attention [18]. As with the activation of appropriate behaviors or thoughts, inhibitory function might also be boosted by a warm-up activity [19]; however, this theory has not yet been directly tested.

**Fig 2:**
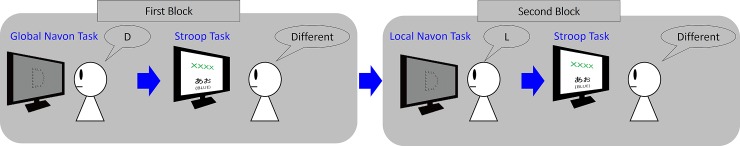
Experimental design. All participants engaged the global condition and the local condition. The order of the conditions was counterbalanced. In each condition, after the Navon task, they completed the Stroop task. Each condition contained either the global or the local Navon task.

**Fig 3:**
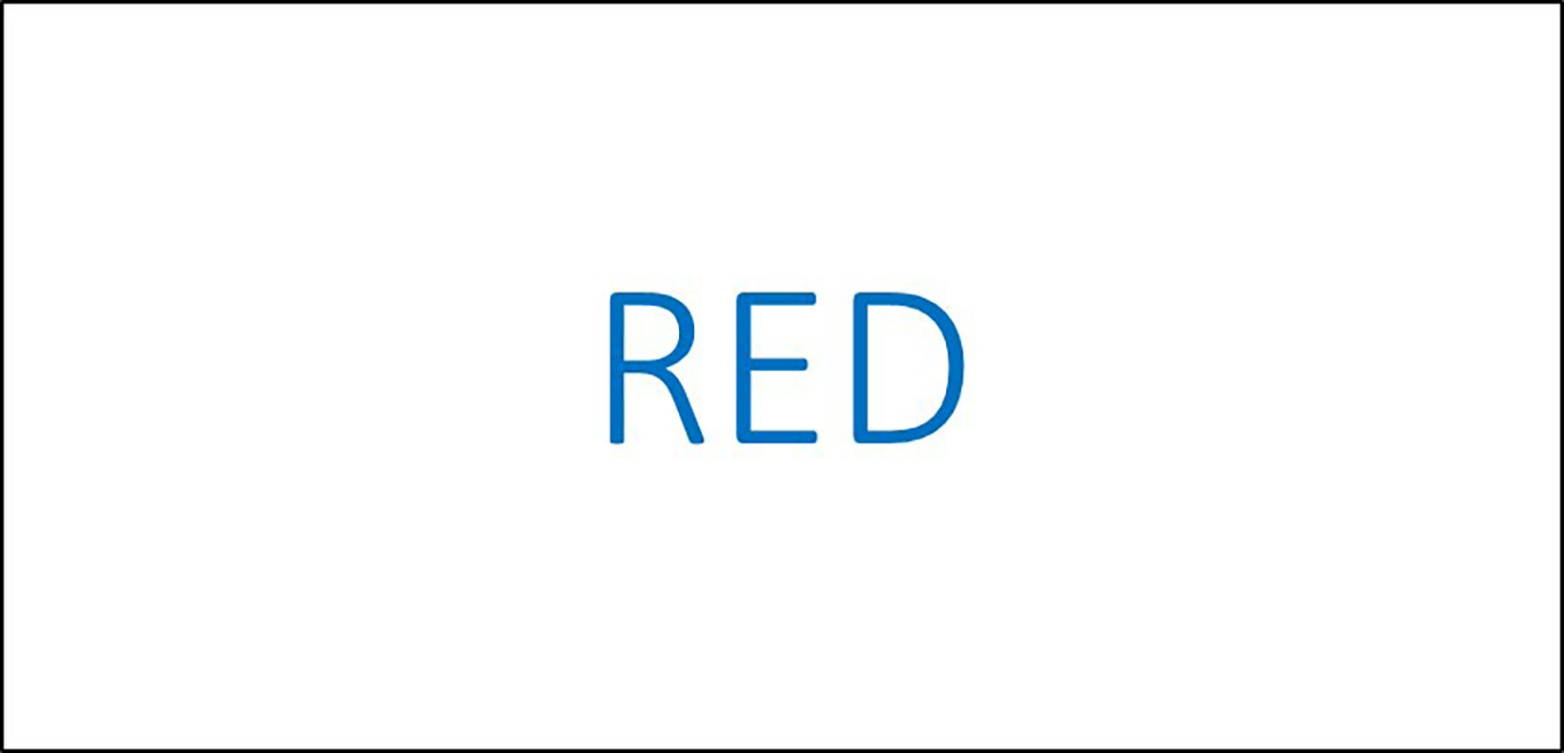
“RED” printed with blue ink. In this task that has the word “RED” printed with blue ink, people have difficulty in reporting the color of the word (blue) when they fail to inhibit reading the word (“RED”).

In the current study, we investigated whether inhibitory function is enhanced by a warm-up activity. To investigate this, we conducted a series of psychological experiments with a Navon task and a Stroop task (Fig 2). We used a Navon task [20] as a warm-up activity task in which a participant was required to read a large letter in a Navon figure (global Navon task; answering “Z” in Fig 1) or small letters (local Navon task; answering “T” in Fig 1). The local Navon task has been reported to require inhibitory function more than the global Navon task because reading a large letter in a Navon figure is an automatic cognitive process [21] and should be suppressed for the local Navon task [22, 23]. The Stroop task [24] was used as the subsequent task. Fig 3 shows an example of the Stroop task. In the Stroop task, naming the color of the ink requires the inhibition of reading the word, and the successful inhibition leads to enhanced performance in the Stroop task. We chose the Stroop task as the subsequent task rather than the Navon task. The Stroop task is one of the most well-established measures of inhibition not only in experimental studies but also in clinical situations [25] and in studies exploring neural mechanisms [26]. The existence of a large literature examining Stroop effects will allow an assessment of whether the inhibitory function is enhanced in the subsequent task because we are able to compare the results of the current study to the results of previous studies. If inhibitory function can be enhanced by an inhibitory warm-up activity, the performance in the Stroop task after the local Navon task is expected to be better than performance after the global Navon task.

## Materials and methods

### Participants

We calculated the sample size with G*Powers [27]. We expected that we would obtain medium effect size values (η^2^ = .12) because a previous study about warm-up activities that used the Navon task showed a medium effect size (η^2^ = .12) [7]. A power analysis of repeated-measures analysis of variance with G*Powers recommended the enrollment of 14 participants corresponding to an effect size f of 0.37 (equivalent to η^2^ = .12), power of 0.80, p value of 0.05, number of groups of 1, number of measurements of 6, correlation among repeated measures of 0.75, and a nonsphericity correction ɛ of 0.2.

Fourteen participants (10 women and 4 men) aged 19–26 years (*M*_*age*_ = 20.2 years) took part in the series of experiments. This study was reviewed and approved by the Ethics Committee of Keio University Faculty of Letters, Graduate School of Letters, and Graduate School of Human Relations. All participants signed the letter of consent.

### Stimulus and procedure

The Navon task and the Stroop task were presented on a 15-inch screen. Each participant performed two blocks of trials (i.e., one global and one local) with both the Navon task and the Stroop task. Each block included 100 trials in the Navon task and 36 trials in the Stroop task. Each block contained either the global or the local Navon task. The order of the blocks was counterbalanced. The Navon task used in the current experiment was the same as that used in the study by Hine and Itoh [7]. The large letter in the Navon figure was always different from the small letters. Each trial began with the presentation of a fixation cross at the center of the screen, followed by a Navon figure. Then, three capital letters were presented that were aligned horizontally. The size of the three capital letters in the global condition was the same as the size of the Navon figure, whereas the size of the three letters in the local condition was the same size as the small capital letters in the Navon figure. For the global condition, participants were required to report the position (i.e., left, center, or right) of the large letter in the Navon figure. For the local condition, participants had to state the position of the small capital letters in the Navon figure. Participants responded by pressing the 1, 2, or 3 on the 10-key number pad (for left, center, and right, respectively). Participants were asked to respond as quickly and correctly as possible. The three capital letters disappeared when they made a response. The interstimulus interval (ISI) was 1000 ms. When a participant chose the wrong answer or the reaction latency was over 600 ms, “×” or “Speed UP”, respectively, was presented as feedback. The Navon task was performed for 3 minutes.

**Fig 4:**
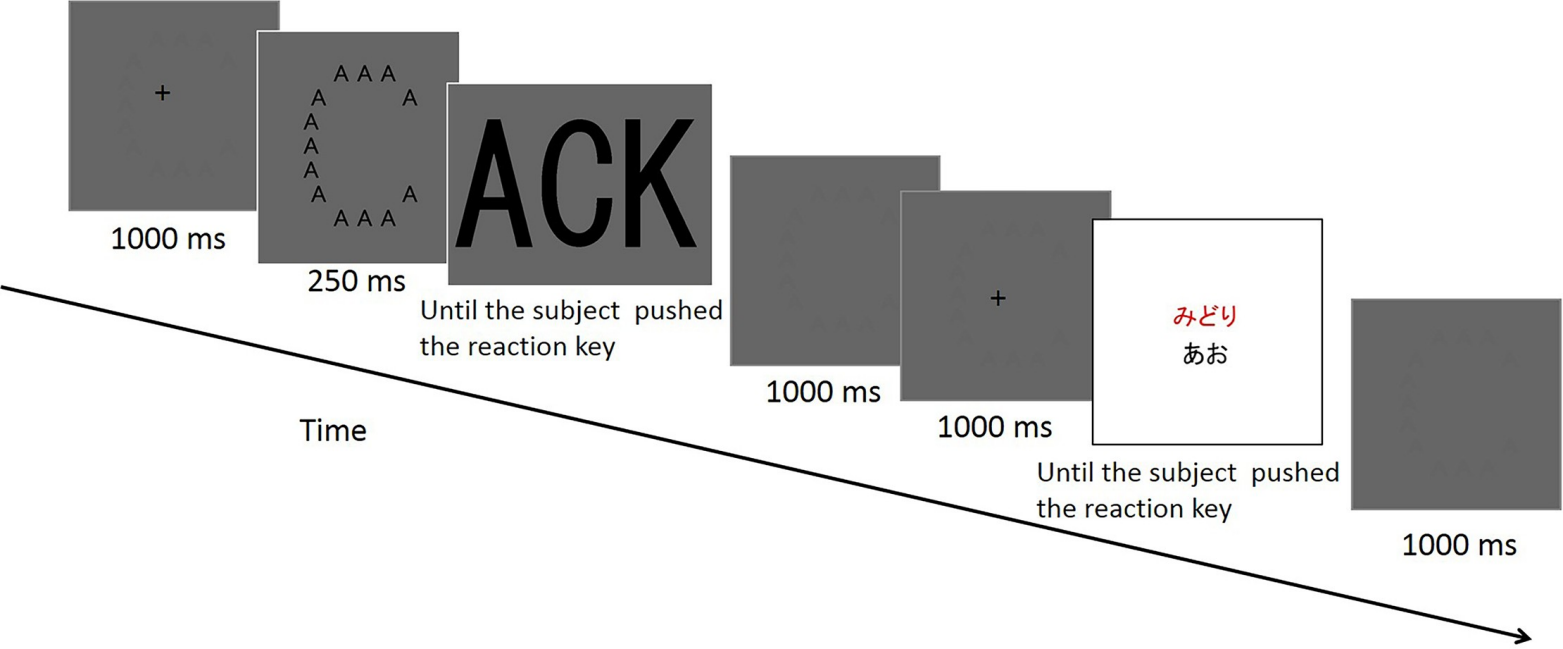
Example of the timeline of one trial in the Stroop task. The size of the three capital letters in the global condition was the same as the size of the Navon figure, whereas the size of the three letters in the local condition was the same as the size of small capital letters in the Navon figure.

**Fig 5:**
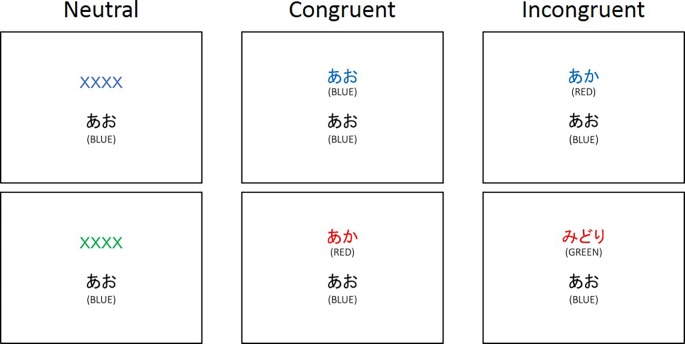
Examples of the stimuli used in the Stroop task. For the top three, the correct answer would be ‘yes’. For the bottom three, the correct answer would be ‘no’.

After the Navon task, participants completed the color-word matching Stroop task [5, 28–31]. Fig 4 shows the timeline of one trial in the Stroop task. At the beginning of each trial of the Stroop task, one trial of the Navon task, which was the same as the 3-minute Navon task, was conducted without feedback. Then, for the Stroop task, two words were presented with one above the other (Fig 4). Participants were required to report whether the color of the ink of the top row was the same as the meaning of the word in the bottom row. Participants responded with 1 or 2 on the number pad (for same and different, respectively). The Stroop task included three conditions (Fig 5). For the neutral condition, the letters in the top row were ‘XXXX’. The color of the ink was red, blue, green, or yellow. For the congruent condition, the word on the top was ‘RED’, ‘BLUE’, ‘GREEN’, or ‘YELLOW’ in Japanese. The color of the word on the top corresponded to the meaning of the word on the top. For the incongruent condition, the word on the top was the same as that for the congruent condition. However, the color of the ink on the top was always different from the meaning of the word. The word on the bottom was ‘RED’, ‘BLUE’, ‘GREEN’, or ‘YELLOW’ in Japanese for the three conditions. For all conditions, the color of the bottom word was always black. To achieve consecutive visual attention [25], the top of the row was presented for 100 ms before the bottom row [28]. There was no feedback on the Stroop task to avoid the decay of the effect of the Navon trial on the Stroop trial by the long ITI [32]. The presentation order was randomized. Participants did not perform the Navon task or the Stroop task in isolation before performing blocks.

## Results

### Accuracy and reaction time on the Navon task

We calculated the accuracy as the rate of correct answers and the reaction time on the Navon task excluding the trials in the Stroop task. The mean rates of accuracy were 0.98 (*SD* = 0.02) for the global Navon task and 0.97 (*SD* = 0.03) for the local Navon task. There was no significant difference, *t*(13) = 0.69, *p =* .500, *r* = .19. The mean reaction times were 463.69 ms (*SD* = 45.78) for the global Navon task and 495.94 ms (*SD* = 29.05) for the local Navon task. The average reaction times for the global Navon task were faster than for the local Navon task, *t*(13) = 3.68, *p* = .003, *r* = .72. We confirmed that the global precedence effect occurred in the current experiment [20].

### Accuracy and reaction time in the Stroop task

**Fig 6:**
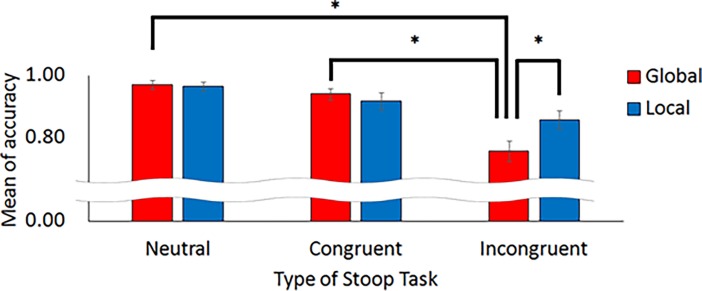
The accuracy on the Stroop task (means ± standard errors).

Fig 6 shows the mean accuracy in the Stroop task. For the global condition, the accuracies in the neutral, congruent, and incongruent conditions were 0.97 (*SD* = 0.05), 0.94 (*SD* = 0.07), and 0.76 (*SD* = 0.12), respectively. For the local condition, the accuracy in the neutral, congruent, and incongruent conditions was 0.96 (*SD* = 0.05), 0.92 (*SD* = 0.11) and 0.86 (*SD* = 0.11), respectively. A two-way ANOVA was conducted on accuracy in the Stroop task with the type of Stroop task (neutral, congruent, incongruent) and Navon task (global, local) as within-subject factors. The main effect of the Stroop task was significant, *F*(2, 26) = 25.21, *MSE* = 0.08, *p* < .001, partial *η*^*2*^ = .36. The main effect of the Navon task was not significant, *F*(1, 13) = 1.03, *MSE* = 0.12, *p =* .329, partial *η*^*2*^ = .01. There was a significant interaction, *F*(2, 26) = 6.86, *MSE* = 0.05, *p* = .004, partial *η*^*2*^ = .06. A simple main effect of the Navon task in the incongruent condition was found, *F*(1, 39) = 10.30, *MSE* = 0.01, *p* = .003, partial *η*^*2*^ = .46. However, a simple main effect of the Navon task was not found in the neutral condition, *F*(1, 39) = 0.04, *MSE* = 0.01, *p =* .851, partial *η*^*2*^ = .00, or in the congruent condition, *F*(1, 39) = 0.57, *MSE* = 0.01, *p =* .455, partial *η*^*2*^ = .05. The simple main effect of the Stroop task was found both in the global condition, *F*(2, 52) = 30.24, *MSE* = 0.01, *p* < .001, partial *η*^*2*^ = .45, and in the local condition, *F*(2, 52) = 6.47, *MSE* = 0.01, *p* = .003, partial *η*^*2*^ = .15. The Bonferroni correction was applied for follow-up analyses. For the global condition, there was a significant difference between the neutral and the incongruent condition and the congruent and the incongruent condition (*p* < .05). For the local condition, there was a significant difference between the neutral and incongruent conditions (*p* < .05).

**Fig 7:**
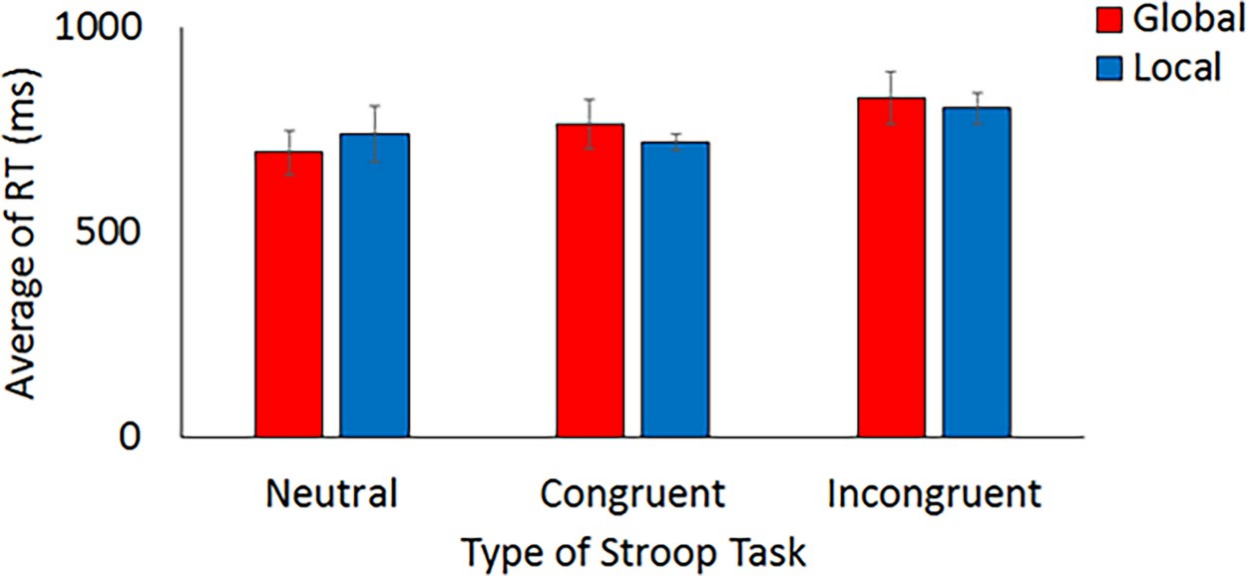
The reaction time on the Stroop task (means ± standard errors).

Fig 7 shows the mean reaction time on the Stroop task. For the global condition, the average reaction time in the neutral, congruent, and incongruent conditions was 695 ms (*SD* = 201), 763 ms (*SD* = 224), and 827 ms (*SD* = 232), respectively. For the local condition, the mean reaction time in the neutral, congruent, and incongruent conditions was 740 ms (*SD* = 253), 719 ms (*SD* = 75), and 802 ms (*SD* = 136), respectively. The main effect of the Stroop task was significant, *F*(2, 26) = 7.76, *MSE* = 9309, *p* = .002, partial *η*^*2*^ = .37. Follow-up analyses with the Bonferroni method were conducted. There was a significant difference between the neutral and the incongruent condition and the congruent and the incongruent condition (*p* < .05). The main effect of the Navon was not significant, *F*(1, 13) = 0.04, *MSE* = 33268, *p =* .846, *η*^*2*^ = .00. The interaction was also not significant, *F*(2, 26) = 1.47, *MSE* = 15264, *p =* .249, *η*^*2*^ = .10. Therefore, a trade-off between accuracy and speed was not found in the current study.

## Discussion

The aim of the current study was to assess whether inhibitory function is enhanced by a warm-up activity. We used the Navon task as the warm-up activity and the Stroop task as the subsequent task. As a result, accuracy on the Stroop task after the local Navon task was higher than after the global Navon task. The local Navon task and the Stroop task require inhibitory function. Therefore, the results of the current study clearly indicate that the warm-up activity boosted the inhibitory function in the subsequent task.

One might assume that the enhancement of the Stroop task in the current study was caused by the size of the target figures in the Navon task. While reading the small letters in the Navon figure, the participant has a narrower attentional focus than when reading the large letter. For the Stroop task, color identification is easy when the focus is sufficiently narrow to not read the words. Therefore, one might think that the small size of the letters in the local Navon task facilitates the subsequent Stroop task. Actually, the performance on the Stroop task after the global Navon task for the incongruent condition was worse than that for the congruent condition, whereas the accuracy after the local Navon task for the incongruent condition was not different from that for the congruent condition. However, if the size of the letters determines the accuracy of the Stroop task, the accuracy in the global condition should be higher than that in the local condition for the congruent condition because the effect of congruency should occur only in the global condition in which participants more easily read the word in the Stroop task. However, this was not observed in the current study. Therefore, the size of the target figures in the Navon task did not significantly determine the main findings in the present study.

Mood might also affect Stroop task performance. Hart et al. reported that performance on an incongruent trial in a negative mood condition was lower than in a neutral mood condition [33]. There is a possibility that the Navon task affected the participants’ mood, and the participants’ mood affected the performance of the Stroop task. In terms of the participants’ mood, a positive mood is known to relate to global processing, whereas a negative mood relates to local processing [34, 35]. Therefore, the performance on the Stroop task after a local Navon task that could induce negative mood would be expected to be lower than the performance after a global Navon task that could induce a positive mood. However, such a result was not found in the present study. Accordingly, mood changes cannot fully explain the findings of the current study, and the outcome still suggests that inhibitory function in the Stroop task was enhanced by the local Navon task.

The results of the current study may also contribute to an understanding of congruency sequence effect [36]. In general, the accuracy of the Stroop task in the congruent condition was higher than that in the incongruent condition. Previous studies showed that the Stroop effect after incongruent trials was reduced compared to the effect after congruent trials, and this has been called congruency sequence effect. Congruency sequence effect is a landmark of conflict adaptation. In the present study, the Navon stimuli in the local Navon task are incongruent stimuli because the small letters never corresponded to the large letters. These stimuli led to making a conflict response. On the other hand, the Navon stimuli in the global Navon task could be regards as congruent stimuli because reading the large letters is a more automatic process than reading the small letters. These stimuli might not be considered a conflict response. If this is the case, the results of the current study could be accounted for by the preceding incongruent stimulus in the Navon task causing the reduction of the Stroop effect. In the past, many studies have investigated what determines whether the congruency sequence effect transfers across different tasks. If the results of the current study could be accounted by the congruency sequence effect, the conflict adaptation was transferred across different tasks. When the congruency sequence effect is found across different tasks, based on the present study, the preceding incongruent stimulus might induce inhibitory processing, and the inhibitory processing transfers to the following task. In future studies, the inhibitory processing by the preceding incongruent stimulus should be assessed to clarify the influence of conflict adaptation.

As mentioned above, it is suggested that the attentional focus induced by the size of the target figures in the Navon task did not determine the results of this study. However, there is still the possibility that the difference between global attentional focus and local attentional focus in the Navon task affected the performance of the subsequent Stroop task. One of the ways to dissociate if the results were due to an enhancement of inhibitory function or due to a difference between a global focus and local focus is to use not only incongruent stimuli but also congruent (the large letter corresponds the small letters) and neutral (only global or local size letter) stimuli during the global and the local Navon tasks. The inhibitory function involved during reading the small letters in the incongruent stimulus would be more than that during reading the small letters in the congruent or neutral stimulus. On the other hand, the inhibitory function involved during reading the large letters in the incongruent stimulus would not be different from that during reading the large letters in the congruent or neutral stimuli. By comparing these results, we could evaluate the effect of inhibitory function and the attentional focus separately. To clarify the determinants of the results of the current study, further studies are needed.

The present research reveals that a warm-up activity enhances the performance of the subsequent cognitive task that required inhibitory function. Regarding traffic accidents by the elderly people, a warm-up activity, such as paying attention to the local features of a complex stimulus may enhance subsequent inhibitory function, and this activity may prevent serious accidents caused by the reduction of the inhibitory function. This consequence might contribute to making up for the decline in cognitive function associated with other clinical conditions such as attention-deficit/hyperactivity disorder (ADHD).
